# m6A methylation modification and immune infiltration analysis in osteonecrosis of the femoral head

**DOI:** 10.1186/s13018-024-04590-6

**Published:** 2024-03-15

**Authors:** Weihua Fang, Peng Peng, Kun Lin, Fangjun Xiao, Wei He, Mincong He, Qiushi Wei

**Affiliations:** 1 Guangzhou University of Chinese Medicine, Guangzhou, China; 2 Guangdong Research Institute for Orthopedics and Traumatology of Chinese Medicine, Guangzhou, China; 3 Department of Orthopaedics, The Third Affiliated Hospital, Guangzhou University of Chinese Medicine, Guangzhou, China

**Keywords:** RNA N6-methyladenosine, Osteonecrosis of the femoral head, Immune microenvironment, Subtype classification, Bioinformatic analysis

## Abstract

Osteonecrosis of the femoral head (ONFH) is a elaborate hip disease characterized by collapse of femoral head and osteoarthritis. RNA N6-methyladenosine (m6A) plays a crucial role in a lot of biological processes within eukaryotic cells. However, the role of m6A in the regulation of ONFH remains unclear. In this study, we identified the m6A regulators in ONFH and performed subtype classification. We identified 7 significantly differentially expressed m6A regulators through the analysis of differences between ONFH and normal samples in the Gene Expression Omnibus (GEO) database. A random forest algorithm was employed to monitor these regulators to assess the risk of developing ONFH. We constructed a nomogram based on these 7 regulators. The decision curve analysis suggested that patients can benefit from the nomogram model. We classified the ONFH samples into two m6A models according to these 7 regulators through consensus clustering algorithm. After that, we evaluated those two m6A patterns using principal component analysis. We assessed the scores of those two m6A patterns and their relationship with immune infiltration. We observed a higher m6A score of type A than that of type B. Finally, we performed a cross-validation of crucial m6A regulatory factors in ONFH using external datasets and femoral head bone samples. In conclusion, we believed that the m6A pattern could provide a novel diagnostic strategy and offer new insights for molecularly targeted therapy of ONFH.

**Supplementary Information:**

The online version contains supplementary material available at 10.1186/s13018-024-04590-6.

## Introduction

Osteonecrosis of the femoral head (ONFH) is a progressive and disabling chronic disease that can cause femoral head collapse, and lead to total hip replacement [1–3]. ONFH is characterized by bone cell necrosis due to ischemia of the femoral head [4]. It is estimated that there are approximately 100,000–200,000 new ONFH cases were diagnosed in China per year [5]. The early symptoms of ONFH are often not apparent, and patients are often diagnosed at the late stages (ARCO stage III–IV). Various approaches exist for treating early ONFH, ranging from surgical interventions to preserve the hip to medication [6–9]. Despite the valuable insights provided by recent studies on ONFH in guiding our clinical practices, a thorough understanding of the molecular mechanism remains elusive. Hence, there is an urgent need to investigate novel biomarkers and effective therapeutic targets for early diagnosis and treatment of ONFH patients.

N6-methyladenosine (m6A) is the most abundant epigenetic RNA modification in eukaryotes, regulating RNA metabolism in cells by affecting writers, erasers, and readers, ultimately impacting human health and disease processes [10]. The m6A function is primarily regulated by the combination of m6A binding protein, demethylase and m6A methyltransferase [11]. M6A is involved in various phases of RNA metabolism, including mRNA translation, degradation, and folding [12]. Dysregulated m6A levels was associated with numerous diseases, such as tumors, neurological disorders, and metabolic conditions [13–15]. Moreover, the m6A RNA methylation also regulates the differentiation of bone marrow mesenchymal stem cells (BMSCs) and involves in the disease progression associated with BMSCs abnormalities [16]. Although studies have confirmed that m6A regulates the translation of various femoral necrosis-related mRNAs in tumor and inflammation-related diseases [17], there is still limited studies detecting the role of m6A in ONFH. Similarly, increasing evidence has highlighted the integral role of inflammatory osteoimmunology in ONFH pathogenesis [18–20]. Therefore, determining the role of m6A and the immune microenvironment in ONFH provides a novel approach for its prevention and treatment.

In this study, we explored the impact of m6A modulators in the diagnostic classification of ONFH and evaluated the degree of immune infiltration in ONFH. We screened seven key regulators from our database to assess the disease risk of ONFH and assessed the immune microenvironment of two m6A patterns of ONFH. Additionally, we conducted cross-validation of key m6A regulatory factors in ONFH using external datasets and bone samples.

## Methods

### Data collection

The ONFH dataset GSE123568 was acquired from the GEO database (https://www.ncbi.nlm.nih.gov/geo/). This dataset comprises peripheral serum samples from 10 healthy individuals and 30 ONFH patients. It contained 25 m6A regulators, consisting of 15 readers, 2 erasers, and 8 writers [21–23].

### Construction of random forest (RF) and support vector machine (SVM) models

We conducted the RF and SVM models to evaluate the risk of ONFH. The RF algorithm was generated by the “randomForest” package in the R software. We determined the importance score > 1, thus screening 13 key regulators out of 25 m6A regulators. SVM is a useful tool for developing predictive models or classifiers [24, 25]. We assessed the accuracy of both models through receiver operating characteristic (ROC) curve analysis, “residual plots,” and “reverse cumulative distribution of residuals.’

### Nomogram model

We created a nomogram model to predict the risk of ONFH using the “RMS” and “rmda” packages in the R software. To evaluate the prediction accuracy of the nomogram model, we utilized decision curve analysis (DCA) and calibration curves. Besides, we used clinical impact curves to determine whether the predictions of the nomogram model would beneficial the patients.

### Identification of two m6A isoforms and m6A gene isoforms

Based on the 25 m6A regulators, the sample data were categorized into m6A models by consistency clustering analysis. Next, we screened the differentially expressed genes (DEGs) related to m6A. After that, we classified the DEGs associated with m6A into two m6A subtypes using consensus clustering analysis. The m6A-related DEGs were implemented by R's “limma” package. For subtype classification, the consensus clustering algorithm was generated by the “ConsensusClusterPlus” package of R software.

### The sample's m6A score

The m6A score was used to assess the genetic characteristics of the m6A pattern of ONFH. The m6A pattern was first determined using principal component analysis (PCA), and then the m6A score was calculated by the following formula: m6A score = 6 (PC1i + PC2i) [26–28].

### Functional enrichment analysis of DEGs between different M6A modes

The gene ontology (GO) enrichment and Kyoto Encyclopedia of Genes and Genomes (KEGG) pathway analysis of DEGs between different M6A patterns were performed. Analysis of GO mainly includes biological process (BP), molecular function (MF), and cellular component (CC) domains [29]. KEGG is a reference knowledge base for the biological interpretation of genomic sequences and other high-throughput data, which enables the identification of potential signaling pathways through KEGG enrichment analysis [30].

### Evaluation of the microenvironment for immune infiltration

In order to investigate the immune infiltration status of ONFH, we performed single-sample gene set enrichment analysis (ssGSEA) to assess the level of immune infiltration in the samples [31]. The gene expression levels of the samples were ranked and their grades were obtained, which were recorded as ssGSEA scores. Then, we detected the degree of immune cell infiltration in each sample.

### Cross-validation of the key m6A regulatory factors in ONFH

We initially confirmed the expression of 7 key m6A regulatory factors in GSE74089. Then, we collected 6 femoral head specimens from the Third Affiliated Hospital of Guangzhou University of Chinese Medicine, comprising three with ONFH and three with femoral neck fractures (FNF). Approval for this study was granted by the Ethics Committee of the Third Affiliated Hospital, and patient informed consent was obtained. The samples were obtained during total hip arthroplasty. The bone tissue was immediately frozen in liquid nitrogen, and RNA extraction followed a standardized protocol. Thermo Scientific's qRT-PCR quantified mRNA transcripts using the NanoDrop Lite and CFX96 Touch real-time PCR detection systems (BIO-RAD, CFX96, USA). Amplification conditions comprised an initial 95 °C for 10 min, succeeded by 40 cycles of 95 °C for 15 s, 60 °C for 30 s, and 60 °C for 30 s. The stability of potential candidate gene expression was evaluated using the standard comparative method (ΔΔCt), with relative target gene expression levels computed using the 2 − ΔΔCt method. Primer sequences are shown in Additional file 1: Supplementary Table 1.

### Statistical analysis

We used linear regression analysis to assess the association between writers and erasers. Additionally, we employed the Wilcoxon test for comparisons between two groups and the Kruskal–Wallis test for multiple group comparisons. Statistical significance was defined as *P* values less than 0.05 (two-sided). All statistical analyses were conducted using R version 4.2.2.

## Result

### Expression of 25 m6A regulators in ONFH

**Fig. 1:**
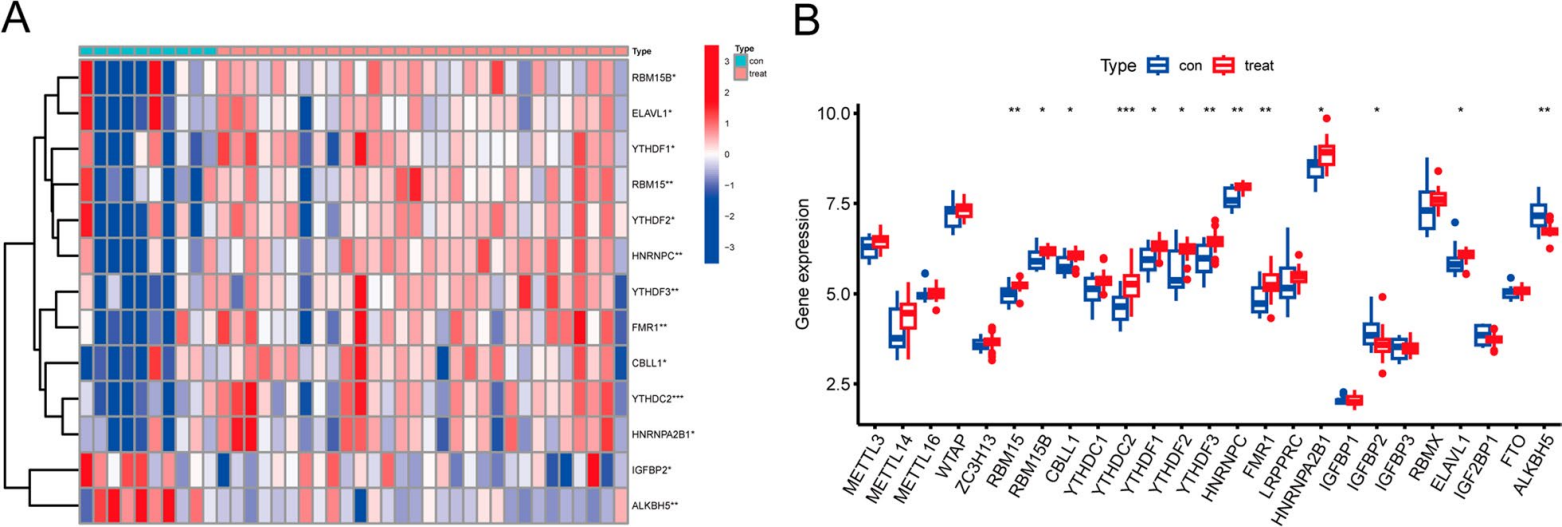
**A** The heatmap of 13 m6A regulators expression in GSE123568. **B** The histogram of 25 m6A regulators expression in GSE123568. *p < 0.05, **p < 0.01, and ***p < 0.001

We performed assessment of the data after removing batch effects. We obtained the expression values of 25 m6A regulators in samples of GSE123568. A total of 13 m6A regulators with significant differential expression (i.e. RBN15, RBM15B, CBLL1, YTHDF1, YTHDF2, YTHDF3, YTHDC2, HNRNPC, HNRNPA2B1, FMR1, IGFBP2, ELAAVL1, and ALKBH5) were identified. The heat map and histogram of 13 differential m6A regulators were shown in Fig. 1A and B, respectively.

### Correlation analysis between writers and erasers

**Fig. 2:**
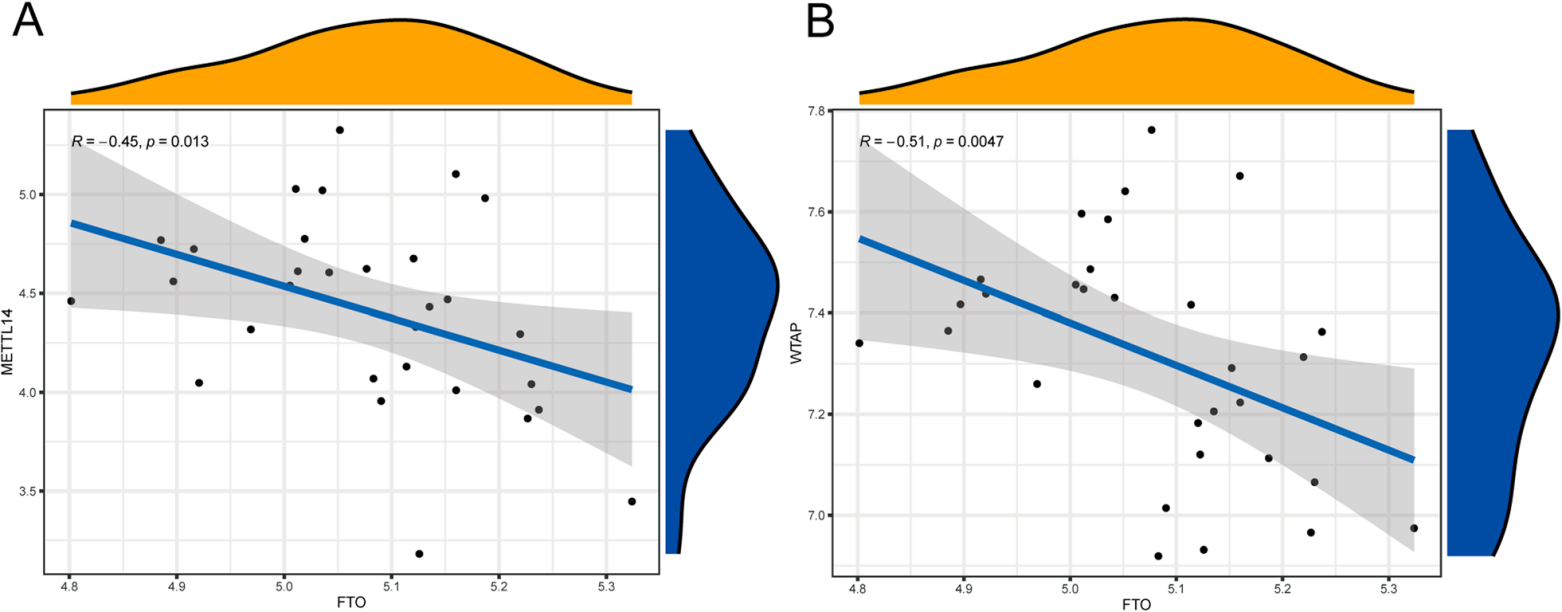
Evaluation of correlation between writers and erasers. **A** A negative association between METTL14 and FTO (*R* =  − 0.45, *p* < 0.05); **B** A negative association between WTAP and FTO (*R* =  − 0.51, *p* < 0.05)

The linear regression analysis revealed that METTL14 was negatively correlated with FTO and ALKBH5 (Fig. 2A), and WTAP was also negatively correlated with FTO and ALKBH5 (Fig. 2B).

### Model selection

**Fig. 3:**
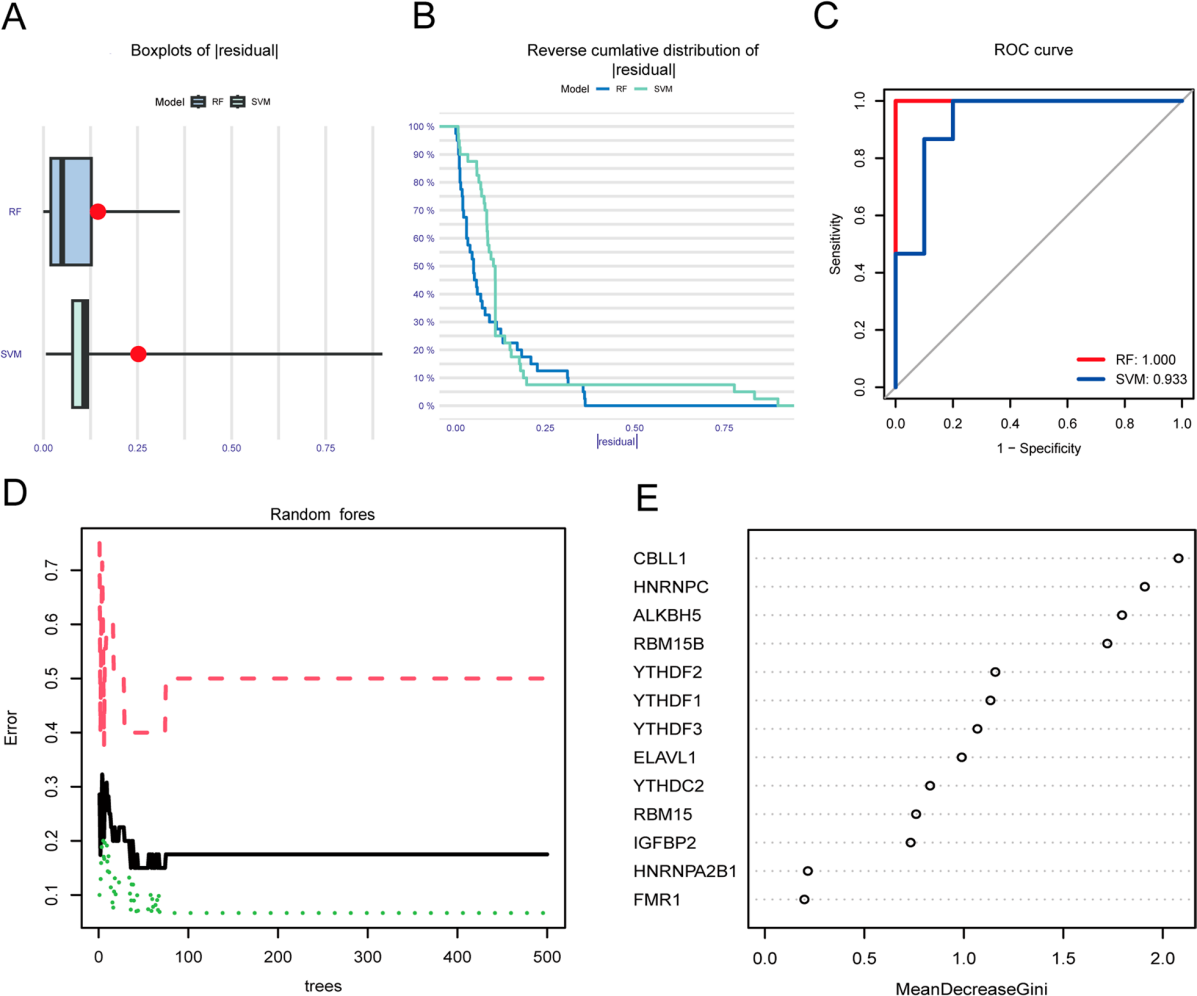
Construction of the RF model. **A** The residuals of SVM and RF models; **B** Boxplots of residual reflecting the residuals of SVM and RF models; **C** ROC curves displayed the accuracy of SVM and RF models; **D** Results of the random forest plot;** E** Significance score of 7 critical m6A moderators

In order to assess the risk of developing ONFH, we developed the RF and SVM models. Compared with the SVM model, the residuals of the RF model are smaller in the “Boxplots of residual” (Fig. 3A) and in the “Reverse cumulative distribution of residual” (Fig. 3B). The results from ROC analysis shows that the RF model has better accuracy (Fig. 3C). Figure 3D shows that the RF model is a good model for predicting the risk of ONFH. As shown in Fig. 3E, the m6A genes were ranked according to the RF model. We screened m6A genes with importance scores > 1 for subsequent disease risk assessment, including CBLL1, HNRNPC, ALKBH5, RBM15B, YTHDF2, YTHDF1, and YTHDF3.

### Construction of the nomogram

**Fig. 4:**
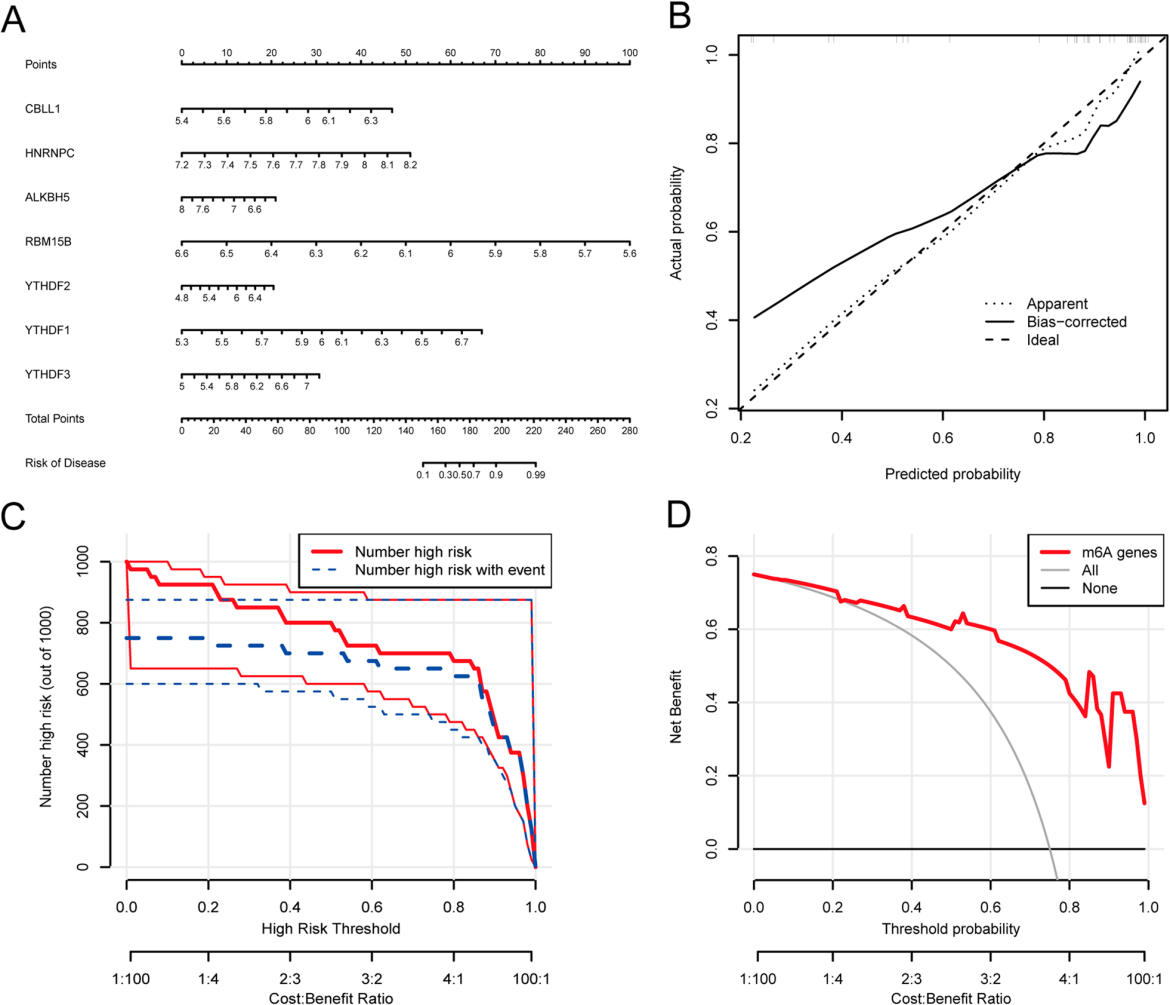
Conduction of a nomogram. **A** Nomogram of 7 critical m6A regulators; **B** Results of calibration curve; **C** The clinical effect for the nomogram model was evaluated with clinical impact curve; **D** The DCA curve showed a high accuracy of the nomogram

We constructed a nomogram model of the 7 key m6A regulators derived from the RF model to evaluate ONFH risk (Fig. 4A). Calibration curves demonstrated the high accuracy of the nomogram model (Fig. 4B). When predicted using the nomogram model, the clinical impact curves showed a high rate of true positives in high-risk patients (Fig. 4C). Decision curve analysis (DCA) curves showed a high predictive accuracy with the nomogram model (Fig. 4D).

### Identification of two isoforms based on 7 key M6A regulators

**Fig. 5:**
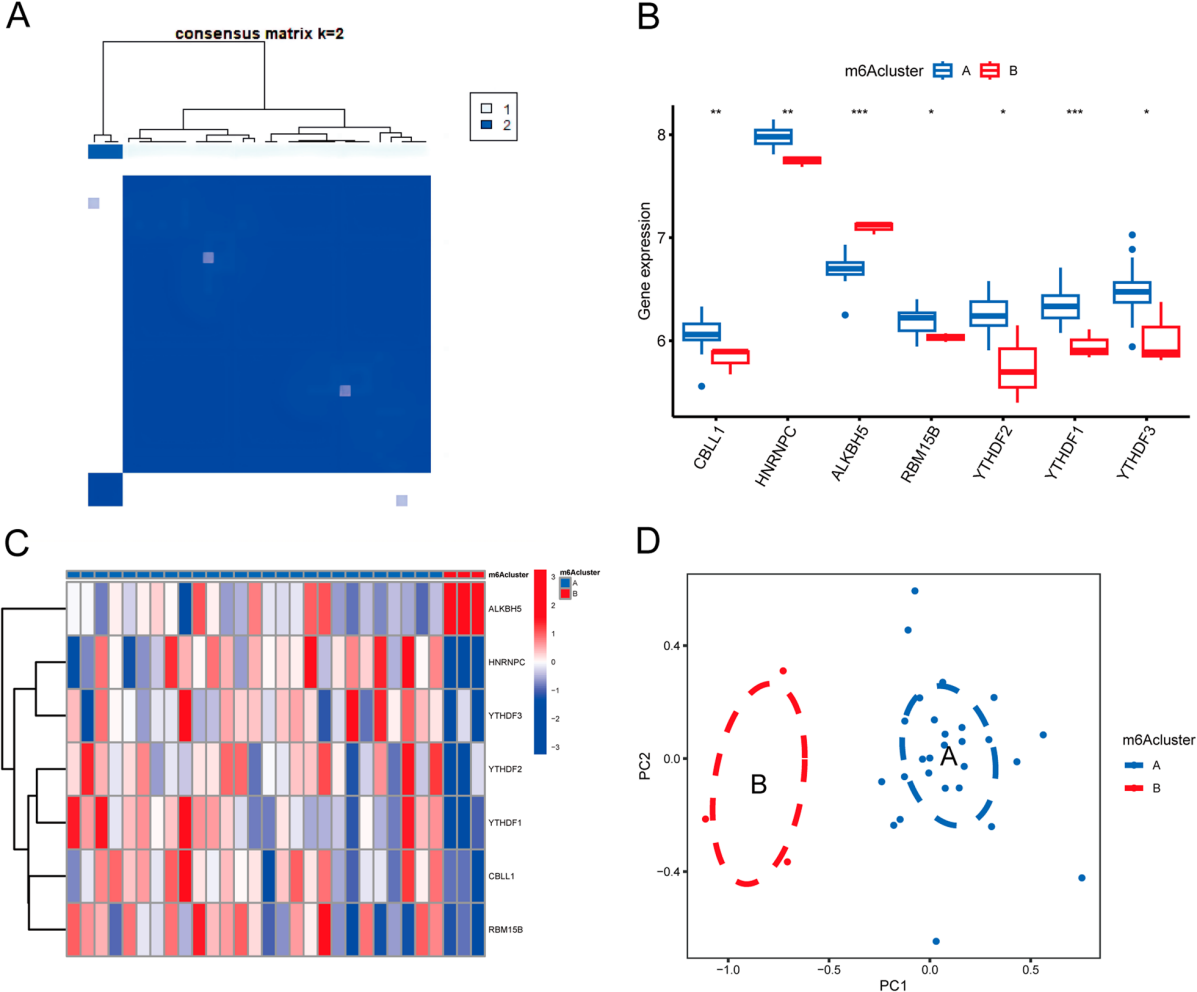
Subgrouping of ONFH samples. **A** Consensus clustering of 7 key regulators; **B** Heatmap of expression of 7 key regulators in the two subgroups; **C** Histogram of expression of 7 key regulators in the two subgroups; **D** PCA of the two subgroup classifications. *p < 0.05, **p < 0.01, and ***p < 0.001

The ONFH samples were classified into two subtypes—m6A cluster A and m6A cluster B. We observed that the consensus was stable when the clusters (*K*) value was 2 (Fig. 5A). The histograms and heatmaps of the expression of the 7 m6A regulators in both subgroups are shown in Fig. 5B and C. These 7 key regulators were further confirmed by PCA to accurately classify ONFH samples into two subgroups (Fig. 5D).

### Investigation of the relationship between immune cell infiltration and M6A subtypes

**Fig. 6:**
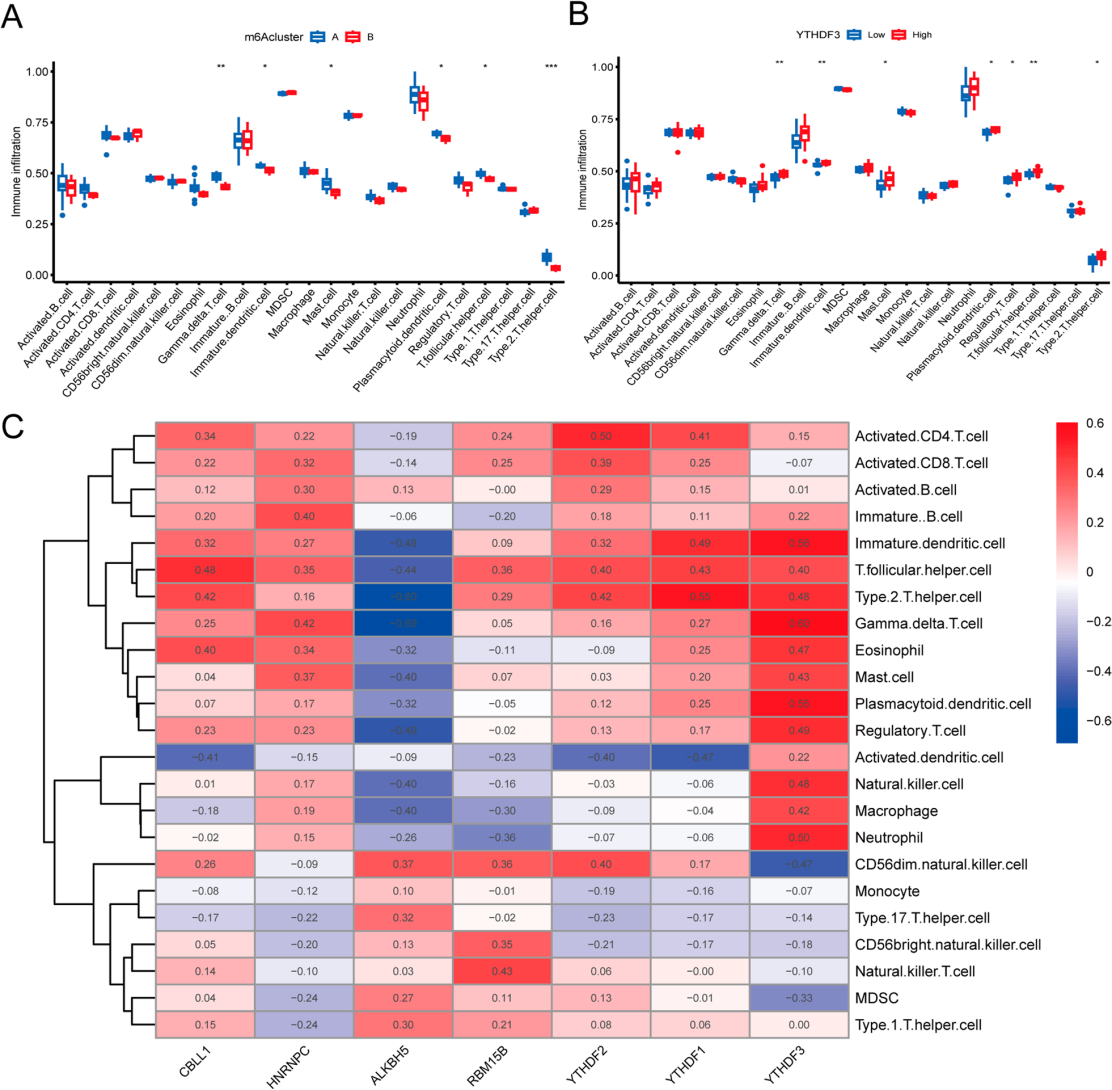
Correlation between immune infltration and m6A. **A** Association analysis of immune infltration and m6A subtypes. **B** Association analysis of immune infltration and m6A genes. **C** Association analysis of immune infltration and 7 key m6A regulators. *p < 0.05, **p < 0.01, and ***p < 0.001

Previous studies reported that the immune inflammatory response generated by immune cell infiltration involved the disease process of ONFH [32, 33]. Therefore, we explored the differences between two m6A subtypes in various immune cells. We found that the two m6A isoforms showed differences in the infiltration of immune cells, including Gamma delta cells, immature dendritic cells, mast cells, plasma cells, T follicular helper cells, and type 2 T helper cells (Fig. 6A). In order to investigate the role of immunity in ONFH, we explored the relationship between 7 key m6A regulators and immune cell infiltration, among which YTHDF3 was the m6A regulator most associated with immune cell infiltration (Fig. 6C). After that, the case samples were divided into YTHDF3 low and high expression groups. There were significant differences in the expression of various immune cells between the YTHDF3 low and high expression groups. The results indicated that these two groups showed significant differences in the infiltration of immune cells, including Gamma delta cells, immature dendritic cells, mast cells, plasma cells, regulatory T cells, T follicular helper cells, and type 2 T helper cells. (Fig. 6B).

### Genotyping based on M6A isoforms

**Fig. 7:**
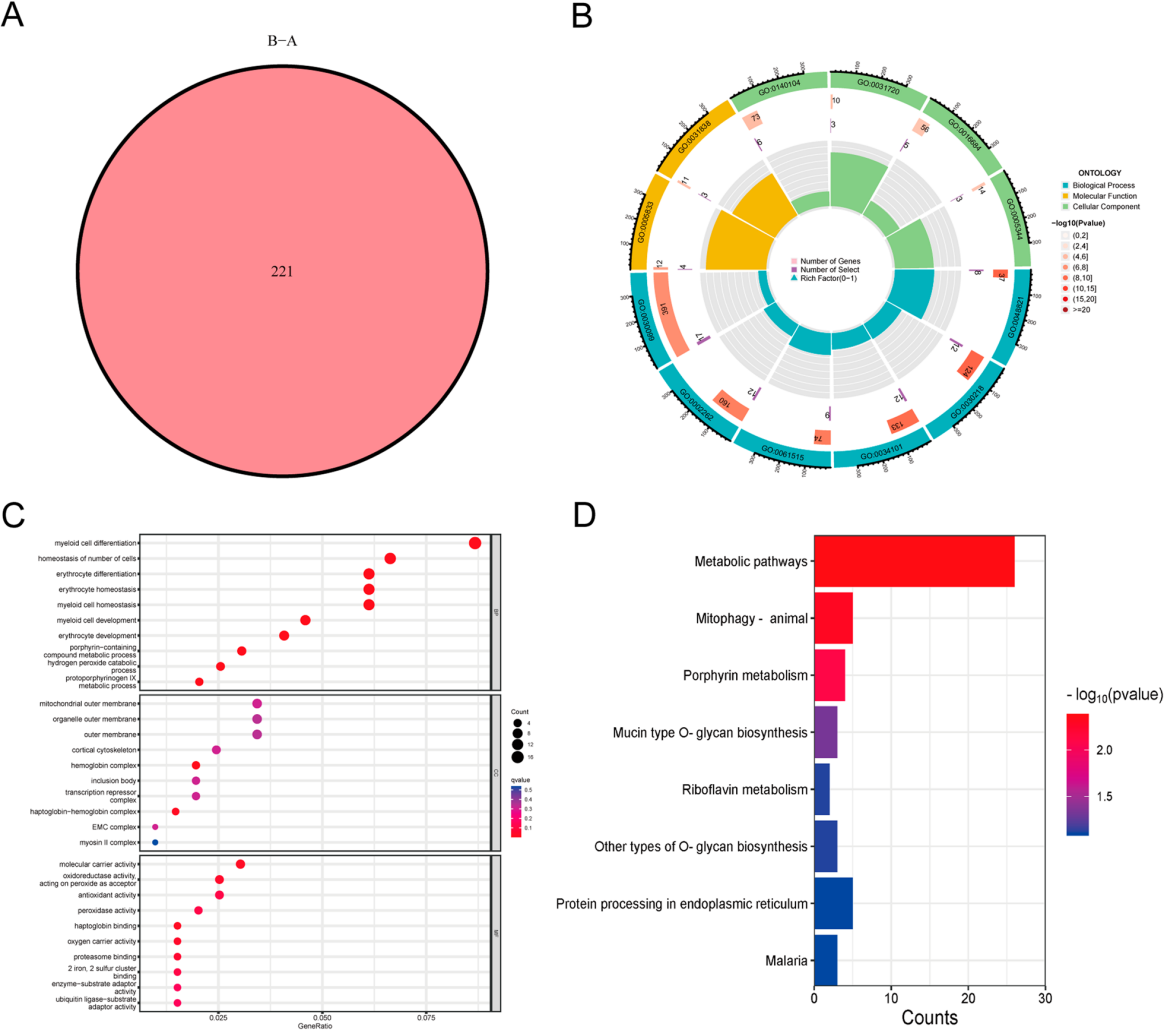
DEGs and GO and KEGG analyses of the m6A subtype. **A** The Venn plot captures the common DEGs of two m6A subtypes; **B** Circular plot presenting GO analysis for the shared DEGs; **C** Bubble plot depicting GO analysis for the DEGs. **D** Barplot displaying the KEGG analysis of DEGs

**Fig. 8:**
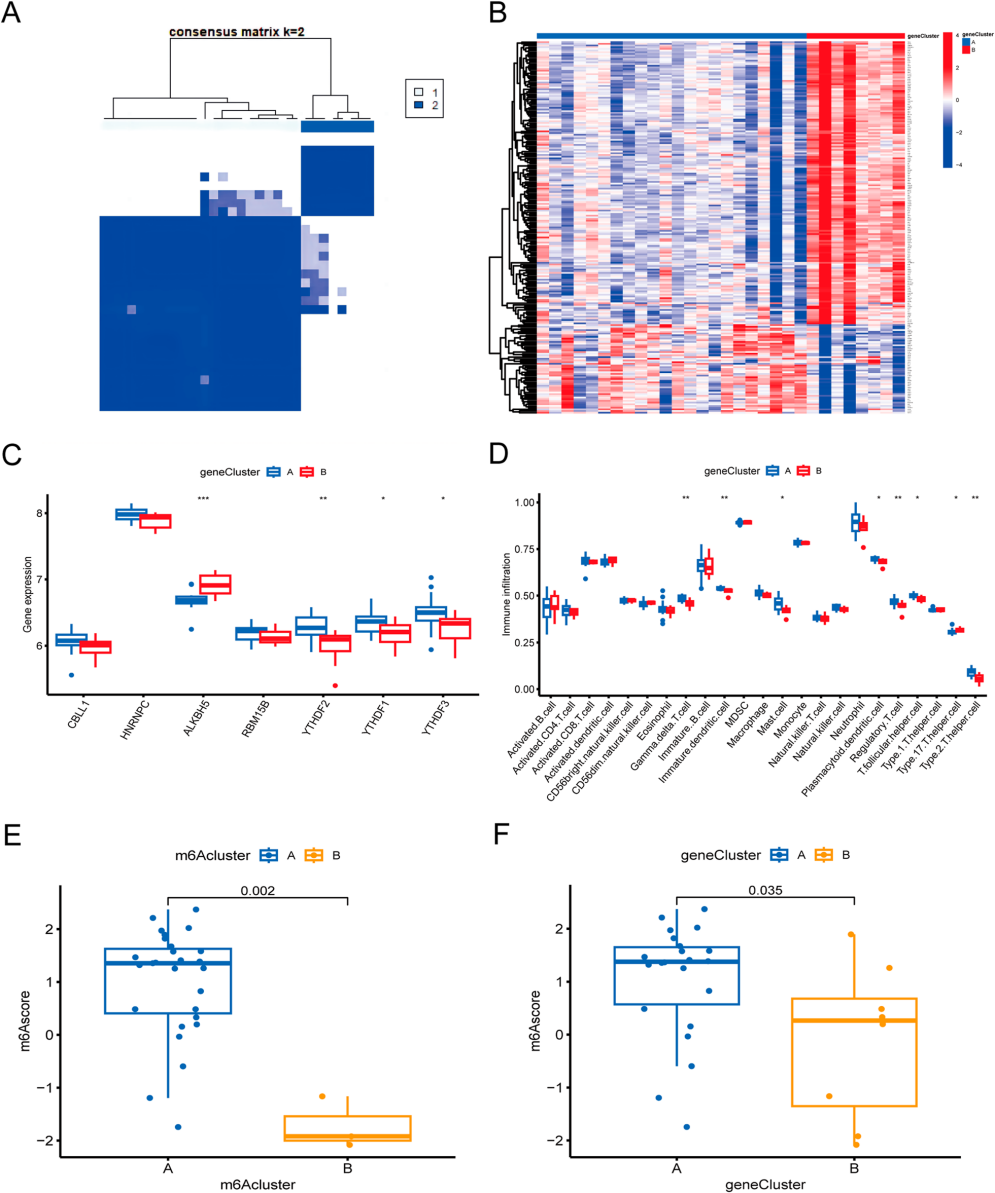
The consensus clustering analysis of DEGs. **A** Consensus clustering analysis divides ONFH patient samples into two genotypes; **B** Heatmap illustrating the differential expression of these 221 genes in two genotypes; **C** Histograms displaying the expression of seven key m6A regulators between two subtypes; **D** Histograms showing the immune infltration between both genotypes; **E** Variations in the m6A scores of both m6A subtypes; **F** Differences in m6A scores between the two subtypes. *p < 0.05, **p < 0.01, and ***p < 0.001

There were 221 DEGs between these two subtypes (Fig. 7A). The GO analysis showed significant enrichment of DEGs for erythrocyte development, hemoglobin complex and molecular carrier activity (Fig. 7B, C). The KEGG analysis was mainly enriched in Metabolic pathways, Porphyrin metabolism and Mucin type O-glycan biosynthesis pathway (Fig. 7B, D). Consist with the m6A subgroup classification, the ONFH samples were classified into two distinct gene subtypes based on 221 DEGs using consensus clustering method. The two genotypes exhibit similar characteristics to the two m6A isoforms (Fig. 8A). The heatmap of expression of 221 DEGs in both genotypes was shown in Fig. 8B. The levels of expression in immune cell infiltration and 7 key m6A regulatory factors are shown as histograms (Fig. 8C, D). We observed that subtype A (m6AclusterA and geneclusterA) of both typing patterns had higher m6A scores than subtype B (m6AclusterB and geneclusterB) (Fig. 8E, F).

### Implications of the m6A model in the diagnosis of ONFH

**Fig. 9:**
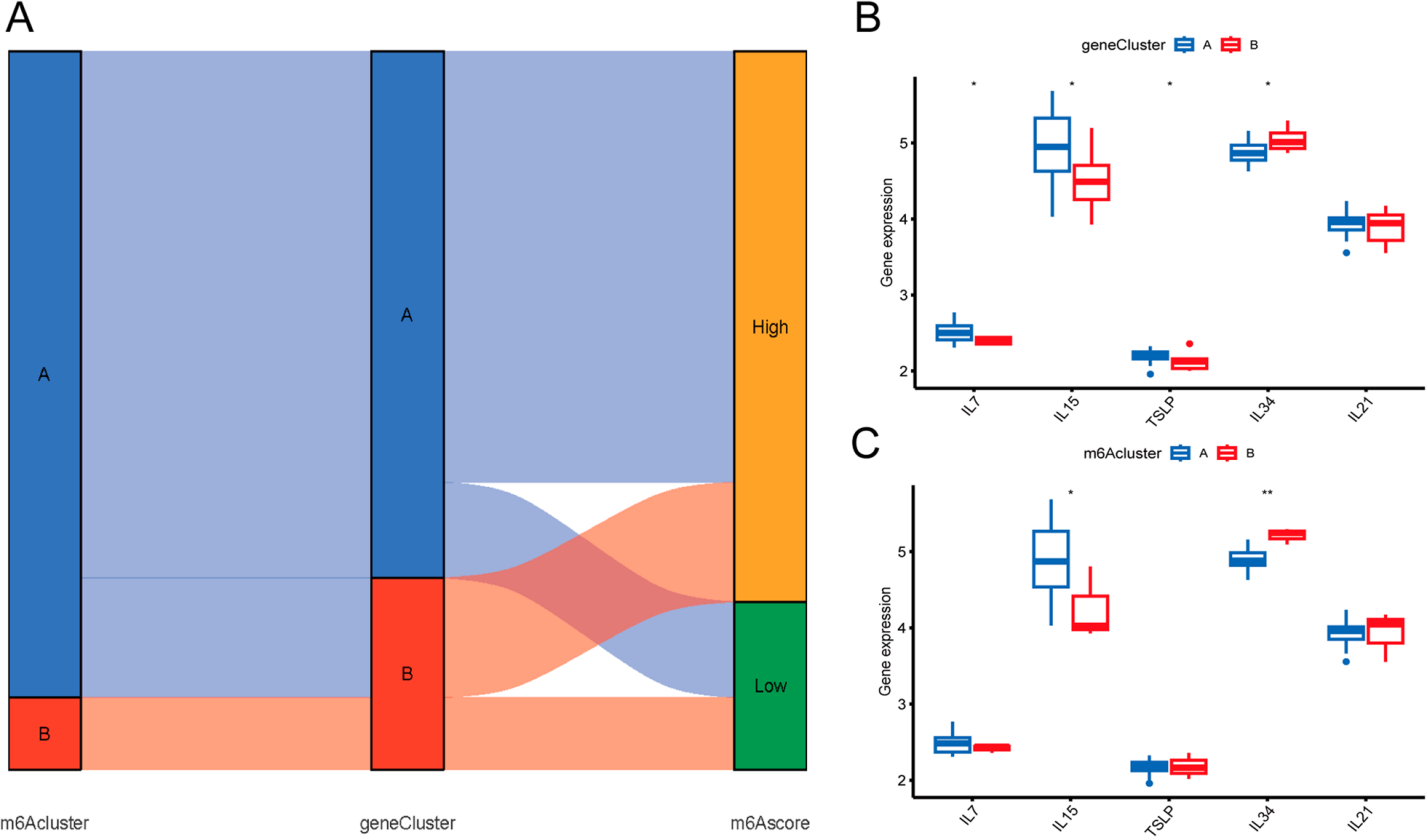
The significance of m6A pattern in ONFH classification. **A** The sankey diagram shows the relationship between the two subtypes; **B** The expression of cytokines in two subtypes; **C** The expression of cytokines in two m6A subtypes

In Sankey plots, m6A subtype A and genotype A had higher m6A scores than that of m6A subtype B and genotype B (Fig. 9A). We analyzed differential expression of cytokines for two gene subtypes (geneclusterA and geneclusterB) (Fig. 9B). We also evaluated the differential expression of some cytokines (IL7, IL15, TSLP, IL34 and IL21) between these two m6A subtype (m6AclusterA and m6AclusterB) (Fig. 9C). In genocluster, IL7, IL15 and TSLP were higher expression in subtype A than that in subtype B, whereas IL34 expression was lower than that in subtype B. In m6Acluster, IL15 was higher expression in subtype A than that in subtype B, whereas IL34 expression was lower than that in subtype B.

### Validation of the key m6A regulatory factors

**Fig. 10:**
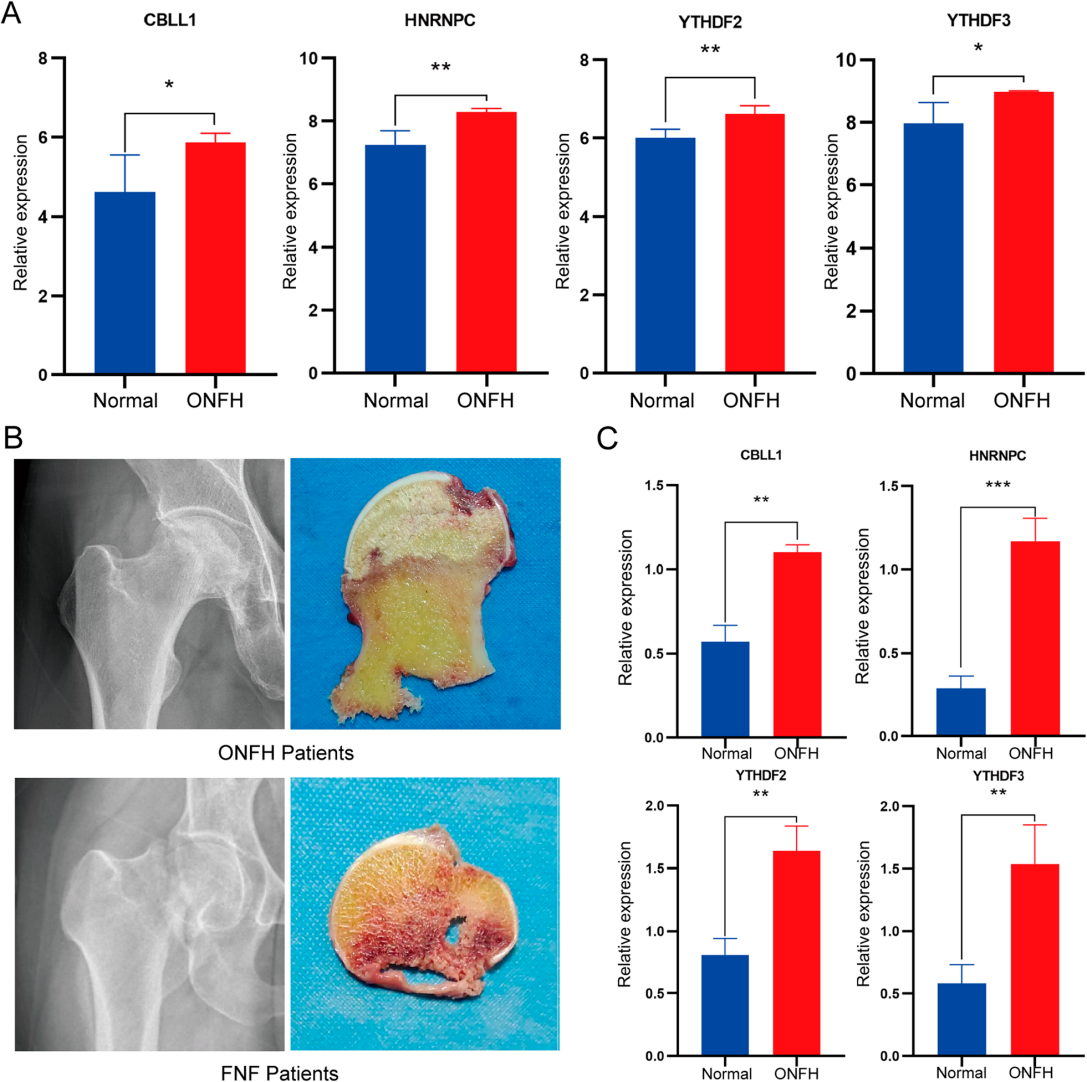
Cross-validation of the key m6A regulatory factors in ONFH. **A** The expression level of m6A regulatory factors in GSE74089. **B** X-ray images of the patient and specimen photographs.** C** The result of qRT-PCR. *p < 0.05; **p < 0.01; ***p < 0.001

Validation using GSE74089 confirmed the congruence of CBLL1, HNRNPC, YTHDF2, and YTHDF3 expression with our study findings (Fig. 10A). In Fig. 10B, we display X-ray images and specimen photos of both the FNF group and the ONFH group patients. We performed qRT-PCR to assess the expression CBLL1, HNRNPC, YTHDF2, and YTHDF3 in bone samples. As shown in Fig. 10C, qRT-PCR analysis indicated a significant upregulation of CBLL1, HNRNPC, YTHDF2, and YTHDF3 expression in ONFH tissues compared to the control group (*P* < 0.05).

## Discussion

ONFH is a chronic degenerative disease with a complex, currently unknown pathomechanism, posing challenges in diagnosis and treatment. Growing evidence indicates that immune response and inflammation play a pivotal role in the pathogenesis of ONFH [34–36]. As the most prevalent mRNA post-transcriptional modification in eukaryotic cells, m6A has garnered significant attention from researchers. The etiology of ONFH is widely recognized to be intricately associated with BMSCs, encompassing factors such as decreased cell quantity and impaired osteogenic potential. In their research, Wang et al. identified that FTO inhibits the osteogenic differentiation of bone marrow mesenchymal stem cells by mediating the demethylation of Runx2 mRNA, consequently leading to a decrease in bone mass [37]. Another study demonstrated that the conditional knockout of METTL3 in BMSCs resulted in increased bone loss and impaired bone formation in mice [16]. Furthermore, the down-regulation of METTL14 expression was observed in ONFH, and its up-regulation was found to enhance the proliferation and osteogenic differentiation of ONFH bone marrow mesenchymal stem cells [17]. These investigations establish a substantial correlation between m6A methylation and the advancement of ONFH. Moreover, recent studies have unveiled its pivotal role in both innate and acquired immune responses [38]. However, the exploration of m6A in patients with ONFH remains limited. In this study, we systematically investigate m6A's modification patterns within the ONFH immune microenvironment. We explore how m6A modifications affect immune cell infiltration, response, function, and activation pathways in ONFH. The finding will provide new insights and guidance for the early diagnosis and target therapy of ONFH.

First, we obtained the transcriptome expression matrix and clinical data of ONFH patients from the GEO database. Through differential expression analysis, we identified 13 differences in m6A gene expression between the normal group and ONFH patients. Specifically, YTHDF3, FMR1, YTHDC2, and RBM15 showed significant upregulation in ONFH samples, while ALKBH5 exhibited significant downregulation in ONFH samples. By utilizing the random forest algorithm, we screened seven key m6A regulators (CBLL1, YTHDF2, ALKBH5, RBM15B, YTHDF1, HNRNPC and YTHDF3) that are implicated in the development of ONFH. Based on these seven key risk factors, we constructed a nomogram model to predict the risk of ONFH. This model holds significance in enabling early identification of individuals at high risk for ONFH during clinical practice, and the identified m6A regulators may serve as potential new molecular targets for ONFH diagnosis or treatment. Furthermore, we validated these 7 key m6A regulators factors through external datasets and bone samples. Furthermore, the precise role and underlying regulatory mechanisms of m6A modification in ONFH remain unclear.

We classified ONFH patients based on the screened 7 key m6A regulators. Additionally, we observed expression correlations between several m6A regulators; for example, METTL14 showed a negative correlation with FTO (*P* = 0.013), which revealed a modeled regulatory network for m6A. We assessed the impact of m6A modification patterns on immune cell infiltration. Our findings indicated a close association between YTHDF3 and the infiltration of inflammatory immune cells in ONFH, such as Gamma Delta cell, immature dendritic cell, and T follicular helper cell. The YTH protein family, characterized by the presence of the YTH structural domain, plays a significant role in organismal development and evolution [39]. According to reports, YTHDF3 plays an important functions in various tumor and immune processes [40]. Moreover, it collaborates with YTHDF1 and YTHDF2 to regulate mRNA stability and degradation, impacting cell proliferation and differentiation [41]. Our study suggests that YTHDF3 may seve a key marker for the regulation of ONFH by immune cells particularly T follicular heloer cell. However, limited research has been conducted on the relationship between YTHDF3 and ONFH. In addition, we found that ONFH immune infiltration was predominantly influenced by immune cells such as Gmma delta cell and Th2 cell under different m6A modifications. T cells, being a key component of cell mediated acquired immunity, are closely associated with the development of ONFH [42]. Th2 cells, direct the immune response through producing signature cytokines to control pathogens and regulate other immune cells [43]. The elevated levels of IL15, IL21, and IL34 in ONFH patients indicate a close association between immune inflammatory responses and ONFH [44–46]. Collectively, these findings suggest that both immune and m6A mediated inflammatory responses play pivotal roles in the ONFH, involving Th2-dominated immune cells and inflammatory mediators.

Our study offers novel insights and guidance for the early diagnosis and target therapy of ONFH. However, certain limitations exist in our research. First, our work in view of bioinformatics analysis, numerous theoretically effective methods require validation through additional wet experiments to confirm their accuracy. Second, the sample size of ONFH patients in our study was limited, warranting a larger cohort for further exploration. Nonetheless, our investigation underscores the substantial influence of m6A modification on ONFH's molecular mechanisms, furnishing a new outlook on comprehending its potential pathogenesis (Additional file 1).

## Conclusion

In conclusion, our study unveils the potential regulatory role of m6A modification within the immune microenvironment of ONFH. These distinctive m6A modification patterns significantly influence the initiation and progression of ONFH by impacting its immune microenvironment. The immunotyping of ONFH patients will assist to design precise immunotherapy strategies for them. Our comprehensive analysis of m6A modification in ONFH enhances the comprehension of its immunomodulatory mechanism, offering valuable insights for treatment and bridging research gaps in this domain.

### Supplementary Information

**Additional file 1.** Supplementary Table 1.

## Data Availability

Data available on request from the authors. The data that support the fundings of this study are available from the corresponding author upon reasonable request.

## Abbreviations

ONFH: Osteonecrosis of the femoral head
m6A: RNA N6-methyladenosine
RF: Random forest
SVM: Support vector machine
DEGs: Differentially expressed genes
GO: Gene ontology
KEGG: Kyoto Encyclopedia of Genes and Genomes
BP: Biological process
MF: Molecular function
CC: Cellular component
ssGSEA: Single-sample gene set enrichment analysis
FNF: Femoral neck fractures
DCA: Decision curve analysis
YTHDF3: YTH N6-Methyladenosine RNA Binding Protein F3
YTHDF2: YTH N6-Methyladenosine RNA Binding Protein F2
CBLL1: Cbl Proto-Oncogene Like 1
HNRNPC: Heterogeneous Nuclear Ribonucleoprotein C

## References

[1] Hao Y, Lu C, Zhang B, Xu Z, Guo H, Zhang G (2021). Identifying the potential differentially expressed miRNAs and mRNAs in osteonecrosis of the femoral head based on integrated analysis. Clin Interv Aging.

[2] Migliorini F, La Padula G, Oliva F, Torsiello E, Hildebrand F, Maffulli N (2022). Operative management of avascular necrosis of the femoral head in skeletally immature patients: a systematic review. Life (Basel).

[3] Quaranta M, Miranda L, Oliva F, Aletto C, Maffulli N (2021). Osteotomies for avascular necrosis of the femoral head. Br Med Bull.

[4] Mont MA, Salem HS, Piuzzi NS, Goodman SB, Jones LC (2020). Nontraumatic osteonecrosis of the femoral head: where do we stand today? A 5-year update. J Bone Jt Surg.

[5] Li M, Zhao Y, Zhang Z, Huang C, Liu Y, Gu J (2020). Chinese Rheumatology Association, National Clinical Research Center for Dermatologic and Immunologic Diseases, Chinese Systemic Lupus Erythematosus Treatment and Research Group. 2020 Chinese guidelines for the diagnosis and treatment of systemic lupus erythematosus. Rheumatol Immunol Res.

[6] Migliorini F, Maffulli N, Baroncini A, Eschweiler J, Tingart M, Betsch M (2023). Prognostic factors in the management of osteonecrosis of the femoral head: a systematic review. Surgeon.

[7] Sadile F, Bernasconi A, Russo S, Maffulli N (2016). Core decompression versus other joint preserving treatments for osteonecrosis of the femoral head: a meta-analysis. Br Med Bull.

[8] Migliorini F, Maffulli N, Eschweiler J, Tingart M, Baroncini A (2021). Core decompression isolated or combined with bone marrow-derived cell therapies for femoral head osteonecrosis. Expert Opin Biol Ther.

[9] He XM, He MC, Yang P, Zhang QW, Chen ZQ, He W (2021). The therapeutic effect of Huo Xue Tong Luo Capsules in Association Research Circulation Osseous (ARCO) stage II osteonecrosis of the femoral head: a clinical study with an average follow-up period of 7.95 Years. Front Pharmacol.

[10] Xu H, Lin C, Yang J, Chen X, Chen Y, Chen J (2022). The role of N^6^-methyladenosine in inflammatory diseases. Oxid Med Cell Longev.

[11] Zaccara S, Ries RJ, Jaffrey SR (2019). Reading, writing and erasing mRNA methylation. Nat Rev Mol Cell Biol.

[12] Liu Q, Gregory RI (2019). RNAmod: an integrated system for the annotation of mRNA modifications. Nucleic Acids Res.

[13] Widagdo J, Anggono V (2018). The m6A-epitranscriptomic signature in neurobiology: from neurodevelopment to brain plasticity. J Neurochem.

[14] Wang S, Chai P, Jia R, Jia R (2018). Novel insights on m6A RNA methylation in tumorigenesis: a double-edged sword. Mol Cancer.

[15] Wei W, Ji X, Guo X, Ji S (2017). Regulatory Role of N6-methyladenosine (m6 A) methylation in RNA processing and human diseases. J Cell Biochem.

[16] Wu Y, Xie L, Wang M, Xiong Q, Guo Y, Liang Y, Li J, Sheng R, Deng P, Wang Y, Zheng R, Jiang Y, Ye L (2018). Mettl3-mediated m6 A RNA methylation regulates the fate of bone marrow mesenchymal stem cells and osteoporosis. Nat Commun.

[17] Cheng C, Zhang H, Zheng J, Jin Y, Wang D, Dai Z (2021). METTL14 benefits the mesenchymal stem cells in patients with steroid-associated osteonecrosis of the femoral head by regulating the m6A level of PTPN6. Aging (Albany NY).

[18] Adapala NS, Yamaguchi R, Phipps M, Aruwajoye O, Kim HKW (2016). Necrotic bone stimulates proinflammatory responses in macrophages through the activation of toll-like receptor 4. Am J Pathol.

[19] Jiang C, Zhou Z, Lin Y, Shan H, Xia W, Yin F (2021). Astragaloside IV ameliorates steroid-induced osteonecrosis of the femoral head by repolarizing the phenotype of pro-inflammatory macrophages. Int Immunopharmacol.

[20] Wang T, Azeddine B, Mah W, Harvey EJ, Rosenblatt D, Séguin C (2019). Osteonecrosis of the femoral head: genetic basis. Int Orthop.

[21] Bai Q, Shi M, Sun X, Lou Q, Peng H, Qu Z (2022). Comprehensive analysis of the m6A-related molecular patterns and diagnostic biomarkers in osteoporosis. Front Endocrinol (Lausanne).

[22] Sendinc E, Shi Y (2023). RNA m6A methylation across the transcriptome. Mol Cell.

[23] Yang J, Wu Z, Wu X, Chen S, Xia X, Zeng J (2022). Constructing and validating of m6a-related genes prognostic signature for stomach adenocarcinoma and immune infiltration: potential biomarkers for predicting the overall survival. Front Oncol.

[24] Sanz H, Valim C, Vegas E, Oller JM, Reverter F (2018). SVM-RFE: selection and visualization of the most relevant features through non-linear kernels. BMC Bioinform.

[25] Huang ML, Hung YH, Lee WM, Li RK, Jiang BR (2014). SVM-RFE based feature selection and Taguchi parameters optimization for multiclass SVM classifier. Sci World J.

[26] Zhang B, Wu Q, Li B, Wang D, Wang L, Zhou YL (2020). m6A regulator-mediated methylation modification patterns and tumor microenvironment infiltration characterization in gastric cancer. Mol Cancer.

[27] Yang Q, Xu F, Jian A, Yu H, Ye T, Hu W (2022). m6A regulator-mediated methylation modification patterns and tumor microenvironment cell-infiltration characterization in head and neck cancer. Front Cell Dev Biol.

[28] Du J, Ji H, Ma S, Jin J, Mi S, Hou K (2021). m6A regulator-mediated methylation modification patterns and characteristics of immunity and stemness in low-grade glioma. Brief Bioinform.

[29] Ashburner M, Ball CA, Blake JA, Botstein D, Butler H, Cherry JM (2000). Gene ontology: tool for the unification of biology. The Gene Ontology Consortium. Nat Genet.

[30] Kanehisa M, Sato Y, Furumichi M, Morishima K, Tanabe M (2019). New approach for understanding genome variations in KEGG. Nucleic Acids Res.

[31] Xue Y, Tong L, LiuAnwei Liu F, Liu A, Zeng S, Xiong Q (2019). Tumor-infiltrating M2 macrophages driven by specific genomic alterations are associated with prognosis in bladder cancer. Oncol Rep.

[32] Dai B, Sun F, Cai X, Li C, Liu H, Shang Y (2021). Significance of RNA N6-methyladenosine regulators in the diagnosis and subtype classification of childhood asthma using the gene expression omnibus database. Front Genet.

[33] Zhang Q, Sun W, Li T, Liu F (2023). Polarization behavior of bone macrophage as well as associated osteoimmunity in glucocorticoid-induced osteonecrosis of the femoral head. J Inflamm Res.

[34] Li T, Huang C, Ma J, Ding R, Zhang Q, Wang W (2022). Identification of inflammation-related genes and exploration of regulatory mechanisms in patients with osteonecrosis of the femoral head. Biomed Res Int.

[35] Jin S, Meng C, He Y, Wang X, Zhang Q, Wang Z, Wang H (2020). Curcumin prevents osteocyte apoptosis by inhibiting M1-type macrophage polarization in mice model of glucocorticoid-associated osteonecrosis of the femoral head. J Orthop Res.

[36] Ma J, Ge J, Gao F, Wang B, Yue D, Sun W (2019). The role of immune regulatory cells in nontraumatic osteonecrosis of the femoral head: a retrospective clinical study. Biomed Res Int.

[37] Wang J, Fu Q, Yang J, Liu JL, Hou SM, Huang X (2021). RNA N6-methyladenosine demethylase FTO promotes osteoporosis through demethylating Runx2 mRNA and inhibiting osteogenic differentiation. Aging (Albany NY).

[38] Zheng Q, Hou J, Zhou Y, Li Z, Cao X (2017). The RNA helicase DDX46 inhibits innate immunity by entrapping m6A-demethylated antiviral transcripts in the nucleus. Nat Immunol.

[39] Ankers JM, Spiller DG, White MR, Harper CV (2008). Spatio-temporal protein dynamics in single living cells. Curr Opin Biotechnol.

[40] Zaccara S, Jaffrey SR (2020). A unified model for the function of YTHDF proteins in regulating m6A-modified mRNA. Cell.

[41] Zuo E, Cai YJ, Li K, Wei Y, Wang BA, Sun Y (2017). One-step generation of complete gene knockout mice and monkeys by CRISPR/Cas9-mediated gene editing with multiple sgRNAs. Cell Res.

[42] Ma M, Tan Z, Li W, Zhang H, Liu Y, Yue C (2022). Osteoimmunology and osteonecrosis of the femoral head. Bone Jt Res.

[43] Nakayama T, Hirahara K, Onodera A, Endo Y, Hosokawa H, Shinoda K (2017). Th2 cells in health and disease. Annu Rev Immunol.

[44] Zhou Z, Lin Y, Pan C, Wang N, Zhou L, Shan H (2020). IL-15 deficiency alleviates steroid-induced osteonecrosis of the femoral head by impact osteoclasts via RANKL-RANK-OPG system. Immun Ageing.

[45] Chen B, Liu Y, Cheng L (2018). IL-21 enhances the degradation of cartilage through the JAK-STAT signaling pathway during osteonecrosis of femoral head cartilage. Inflammation.

[46] Wang F, Min HS, Shan H, Yin F, Jiang C, Zong Y (2022). IL-34 aggravates steroid-induced osteonecrosis of the femoral head via promoting osteoclast differentiation. Immune Netw.

