# Supplementary material for: m6A methylation modification and immune infiltration analysis in osteonecrosis of the femoral head
Source: J Orthop Surg Res. 2024 Mar 15;19:183. doi: 10.1186/s13018-024-04590-6 (PMC10943872; doi:10.1186/s13018-024-04590-6)
Supplement: Supplementary file 1 — Additional file 1. Supplementary Table 1. [file 13018_2024_4590_MOESM1_ESM.docx]

**HNRNPC**  Forward Primer TCCTCCTCCTATTGCTCGGG

Reverse Primer GTGTTTCCTGATACACGCTGA

**YTHDF2**  Forward Primer AGCCCCACTTCCTACCAGATG

Reverse Primer TGAGAACTGTTATTTCCCCATGC

**CBLL1**  Forward Primer TCCTTGGGTGGTCTTGATGTT

Reverse Primer CAGGTTTCGCTTTGTTTGCTT

**YTHDF3** Forward Primer TCAGAGTAACAGCTATCCACCA

Reverse Primer GGTTGTCAGATATGGCATAGGCT
